# Gender differences in the risk of depressive disorders following the loss of a young child: a nationwide population-based longitudinal study

**DOI:** 10.1186/s12888-021-03421-w

**Published:** 2021-08-20

**Authors:** Hsin-Hung Chen, I-An Wang, Shao-You Fang, Yiing-Jenq Chou, Chuan-Yu Chen

**Affiliations:** 1 Division of Pediatric Neurosurgery, The Neurological Institute, Taipei Veterans General Hospital, Taipei, Taiwan; 2 Institute of Public Health, National Yang Ming Chiao Tung University, No. 155, Sec. 2, St. Linong, Taipei City, Taiwan 112; 3 Center of Neuropsychiatric Center, National Health Research Institutes, Zhunan, Taiwan

**Keywords:** Death of a child, Major depressive disorder, Bereavement effects, Treatment seeking

## Abstract

**Background:**

Losing a child to death is one of the most stressful life events experienced in adulthood. The aim of the current study is to investigate parental risk of seeking treatment for major depression disorders (MDD) after a child’s death and to explore whether such connection may operate differentially by parents’ prior medical condition.

**Methods:**

We studied a retrospective cohort of 7245 parents (2987 mothers and 4258 fathers) identified in the National Health Insurance Research Database of Taiwan (NHIRD) who had lost a child with age between 1 and 12 years. For comparison, the parents of 1:4 birth year- and gender-matched non-deceased children were retrieved (16,512 mothers and 17,753 fathers). Gender-specific Cox regression analyses were performed to estimate risk.

**Results:**

Nearly 5.0% and 2.4% of bereaved mothers and fathers sought treatment for MDD within three years after a child’s death, significantly higher than 0.8% and 0.5% in the non-bereaved parents. With covariate adjustment, the hazard ratio (HR) for maternal and paternal seeking treatment for MDD was estimated 4.71 (95% confidence interval [CI]: 3.35–6.64) and 1.93 (95% CI: 1.27–2.95), respectively. The increased risk of MDD varied by prior disease history; specifically, the increased risk of seeking treatment for MDD was especially prominent for those without chronic physical condition (CPC) (e.g., mothers with CPC: aHR = 2.38, 95% CI: 1.56–3.65 vs. no CPC: aHR = 9.55, 95% CI: 6.17–14.79).

**Conclusions:**

After the death of a child, parental elevated risk of MDD was especially prominent for the women and those without prior medical condition. Effective strategies addressing bereavement may require family-based, integrated physical and mental healthcare and even extended counseling service.

**Supplementary Information:**

The online version contains supplementary material available at 10.1186/s12888-021-03421-w.

## Background

Major depressive disorder (MDD), a leading cause of economic burden in many parts of the world, accounted for 8.2% of global years lived with disability in 2010 [1]. A recent review indicated that approximately 4.7% of the world population currently has MDD, and the prevalence varied widely across region and demographic stratum [2]. In Taiwan, the incidence of treated MDD was on the notable rise over the past decades. For the individuals aged 45 to 54 years — the age group with the incidence peak, the estimated rate for men and women was 2.15 and 4.51 per 1, 000 person-year, respectively [3]. Cumulative evidence has pointed out several individual-level health and social characteristics associated with increased risk of onset and recurrence of MDD, including chronic diseases (both mental and physical one), disadvantaged socioeconomic status, low social integration, and stressful life events [4–8].

Having a loved one die was the most frequently reported life event in adulthood [9]. Experiencing the death of a beloved one (especially a family member) often causes extremely complicated persistent grief despite coping mechanisms [10]. An increased risk of adverse health outcomes has been reported among the bereaved spouses, parents, and children [11–13], with the severity of bereavement often depending on the closeness and bonding between the living and the dead. Since the parent-child relationship has been regarded as one of the closest ties that a middle-aged individual may have, the grief after losing a child is often intense and chronic. Studies have consistently demonstrated that the bereaved parents may experience greater physiological and psychological disorders, including cancer, diabetes, depression, anxiety, suicidal attempts, and even death through health-compromising behaviors, dysregulated neuroendocrine/immune systems, and intertwined connections with prior vulnerabilities [14–17]. The emergence of these disorders not only fluctuates over time but also varies by children’s characteristics (e.g., age and unexpectedness of death) [7, 18, 19]. In the case of suicide, the risk was reported to emerge abruptly in the first months of child loss and appeared more salient if the child died in early childhood [20, 21].

For parents, several characteristics have been documented to explain different risks of psychological distress after losing a child [5, 18, 22]. Gender is one attribute of great relevance in identifying possible etiological processes underlying bereavement effects [15, 21, 23, 24]. With few exceptions [25], studies have generally indicated that women were more likely to experience suicidal ideation and attempts, anxiety, depression, lower quality of life, and reduced wellbeing after losing a child as compared with men [18, 19, 21]. Such gender differences in bereavement effects may result from biological vulnerability, social role expectation, and coping strategies [19, 25–27]. Some evidence indicated that in response to life events or stressors, women appeared to use emotional coping styles more often and have their emotions expressive or confrontive. Men, on the other hand, were more likely to use detachment coping styles and have more emotional inhibition [25, 28].

Losing a child to death may exert detrimental effects on parents differentially in terms of observational interval and outcome indicators [14, 19, 22, 25, 29]. Evidence from qualitative interviews and quantitative analyses consistently showed that the bereaved parents mostly took 3–4 years or even longer to put the child’s death into perspective [22, 29]. A recent study analyzing 902 bereaved parents identified from the registry datasets in Finland found that the bereavement effects, as manifested in the use of psychotropic medications, reached the highest prevalence within the first year and fell sharply thereafter in the following three years. For children who died from diseases (not from external causes), the use of psychotropic medication was gradually elevated in the three years preceding the death of a child [22]. Meanwhile, a number of studies have noted that adverse health outcomes subsequent to a child loss may depend on subgroups defined by household characteristics, such as income status, household size, and social network [5, 8].

Although previous studies have highlighted some factors relevant to psychological distress of losing a child, the evidence was mostly collected in Western societies [25]. In traditional East Asian culture, son is more important and preferred for not only economic reasons but his ability to continue the family lineage [19]. In this context, some evidence has supported that physical and psychological distress in the bereaved parents appeared stronger after losing a son than a daughter [19, 30, 31]. Meanwhile, mothers were often the ones taking care of the demand posed by homemaking and childcare, and children take on great symbolic importance on married women’s identity. Finally, these studies often built upon a relatively small sample size and cannot systematically characterize subgroup variation in the manifestation of parental bereavement. In this study, we turned to the National Health Insurance data in Taiwan to identify a retrospective cohort of parents who lost their young children to death, following up throughout three years. Here, with a focus on “MDD” —the most common and disabling mental disorder in middle adulthood, the present study aimed to (i) evaluate father- and mother-specific risk of MDD treatment seeking after a child’s death, and (ii) to investigate whether such MDD risk may be manifested differentially by parents’ prior health condition.

## Methods

### Data sources

Data for the present study came from the 1998–2013 National Health Institute Research Data (NHIRD), derived from the National Health Insurance Plan (NHIP) in Taiwan. The NHIP is a mandatory, single-payer health insurance for all citizens and legal residents, providing healthcare in outpatient, ambulatory, and inpatient service systems. Implemented since 1995, the estimated coverage rate reached 99.5% of the population by the end of 2005. In the NHIP, patients with “catastrophic illness”, defined as severe illness requiring prolonged hospitalization or treatment, will receive the certificates to exempt them from copayments.

### Study design

The present study used a retrospective matched cohort study, and the exposure event is losing a child (i.e., death). In Taiwan, the majority of children who died between age of 12 to 18 years were due to unnatural causes (e.g., accidents and suicides), of which survival status cannot be validated by the National Health Research Dataset. To ensure the exposure status, the present study decided to focus on a subgroup of young individuals who died no older than 12 years during the year from 2002 to 2010 (see Supplementary Fig. 1) (*n* = 10,926). The records concerning death were identified and validated through three sources: insurance status (i.e., withdrawal due to death), treatment records for disease listed as among the 10 leading causes of death for children, and no healthcare records after insurance withdrawal. For comparative purpose, we obtained the children age under 12 who were alive as of the end of 2010 (*n* = 5,809,757). The linkage rate between the index child with at least one parent was 91% for the deceased group, and such rate in the comparison group was 96%. Since for reimbursement purposes, medical treatments for severe or congenital diseases were often recorded in the mother’s healthcare insurance account before the infants had their own individual identification numbers, the present study therefore decided to focus on children who died after reaching their first birthday (*n* = 5881); and their age- and gender-matched living peers were further randomly selected in a ratio of 1:4 (*n* = 23,524). For the deceased children, the linkage rate with fathers and mothers was accordingly 72.4% and 50.8%, respectively, slightly lower than the corresponding estimate for the living children (i.e., 75.5% and 70.2%, respectively).

### Measures

#### Outcome variable

Medical service for MDD (including major depression and any depressive disorder) was defined by the International Classification of Disease, Ninth Revision, Clinical Modification [ICD-9-CM] (296.2X, 296.3X, 300.4X, and 311), given by a board certificated psychiatrist during at least two outpatient services or one inpatient service [32, 33]. The time to the MDD treatment was obtained from the first visit within three years of the index children’s death date.

#### Other variables

Children’s characteristics included gender, age, and duration of the catastrophic illness (i.e., non, less than one year, and one year or longer). Family characteristics included economic status (i.e., income) and residential urbanicity (i.e., urban vs. rural region); linkage with the other parent other than the present insurance payer was obtained to reflect household variation in employment history since the birth of the index child — a proxy measure for parents’ market participation [34]. Mother/father characteristics included age at the death of the index child and premium payer (a proxy for labor market participation). History of chronic physical condition (CPC) (i.e., malignant neoplasms [ICD 9-CM: 140–208], chronic liver disease and cirrhosis [ICD 9-CM: 571], heart disease [ICD 9-CM: 390–392, 393–398, 410–414, 420–429], cerebrovascular disease [ICD 9-CM: 430–438], diabetes [ICD 9-CM: 250], hypertensive disease [ICD 9-CM: 401–405], bronchitis [ICD 9-CM: 490–491], emphysema [ICD 9-CM: 492], asthma [ICD 9-CM: 493], and atherosclerosis [ICD 9-CM: 440]), together with major depressive disorders ([ICD-9-CM: 296.2X, 296.3X, 300.4X, and 311]), were retrieved in the three years before the death of the index child.

### Statistical analyses

**Fig. 1:**
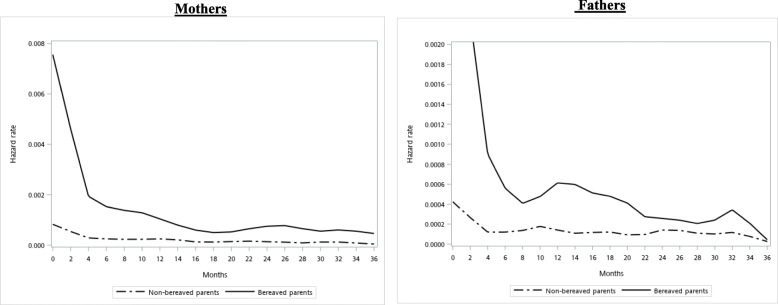
Smoothed hazard estimates of seeking treatment for major depressive disorders in the mothers (left panel) and fathers (right panel) by losing a child

Cross-tabulation was first conducted to summarize selected parents’ and children’s characteristics with stratification by exposure (i.e., losing a young child to death), with differences being evaluated by Chi-square test. Next, the hazard of doctor visit for MDD was estimated by a child’s death and the smoothed hazard function was depicted in the Fig. 1. The time to event was initially calculated as months after the index child’s death till the occurrence of receiving the first diagnosis of MDD, death (i.e., parent’s insurance withdrawal due to death), 36 months, or the end of observation (December 31, 2013), depending on which came first. Further inspection on the log-negative-log of the survival function revealed a parallel in the status of losing a child, indicating that the proportional hazard assumption was held (see the Supplementary Fig. 2). Therefore, we decided to use the Cox proportional hazard regressions to estimate the risk of having received treatment for MDD over the course of three years.

With stratification by parental gender, we first obtained crude hazard ratio of losing a child with parents’ MDD, followed by simultaneous adjustment for child, family, and parental characteristics (Models I and II). The risk was presented in crude and adjusted hazard ratios (HR), with 95% confidence intervals (CI) as an aid for interpretation (see the Supplementary Fig. 3 for the Schoenfeld residuals plot). Finally, potential heterogeneity in bereavement effects was also probed by adding interaction with parents’ previous health condition (i.e., CPC and MDD) (see the Supplementary Fig. 4 for the hazards sub-grouped by prior history of CPC and MDD). A *p*-value less than 0.05 was considered statistically significant. All analyses were performed using SAS version 9.4 (SAS institute, Cary, NC, USA).

## Results

Among those deceased children, nearly one third (*n* = 1861; 31.6%) had a catastrophic illness for more than 1 year compared to 1.4% (*n* = 320) in their birth-year- and gender-matched peers (see Table 1). Approximately 34% of deceased children’s health insurance was covered by their mothers (i.e., the mother had a job), significantly lower than the 44% in their counterparts. In general, the parents of deceased children were more likely to be older (e.g., mothers: 35 years old or older: 44% vs. 42%), to have prior history of MDD (e.g., fathers: 1.83% vs. 0.28%), and to have CPC (e.g., fathers: 39% vs. 12%). Nearly 5 % of bereaved mothers and two-point-4 % of bereaved fathers have sought treatment for MDD within three years after losing a child to death.

**Table 1:** Characteristics of children who died and paired cohort with their parents

Characteristics	Deceased children		Non-deceased children		*P*-value
	n	%	n	%	
Total	5881	100.00	23,524	100.00	
**Child’s and family characteristics**					
Gender					1.0000
Male	3262	55.47	13,048	55.47	
Female	2619	44.53	10,476	44.53	
Age at death					1.0000
1–2 years	1792	30.47	7168	30.47	
3–6 years	2250	38.26	9000	38.26	
7–12 years	1839	31.27	7356	31.27	
Diagnosis of catastrophic illness before death					< 0.001
Less than one year	856	14.56	162	0.69	
One or more years	1861	31.64	320	1.36	
No	3164	53.80	23,042	97.95	
Income level^b^					< 0.001
Poverty	1951	33.17	3898	16.57	
Medium	2448	41.63	11,909	50.32	
High	1257	21.37	7087	30.13	
Missing	225	3.83	630	2.68	
Urbanization^b^					< 0.001
Urban	1515	25.76	6448	27.41	
Suburban	3614	61.45	14,561	61.90	
Rural	650	11.05	2054	8.73	
Missing	102	1.73	461	1.96	
Premium payer^b^					< 0.001
Father	3247	55.21	12,268	52.15	
Mother	2032	34.55	10,354	44.01	
Other^a^	488	8.30	378	1.61	
Missing	114	1.94	524	2.23	
**Maternal characteristics (*N*** **= 19,499)**					
Maternal age at child’s death					0.009
< 35 years	1667	55.81	9633	58.34	
≥ 35 years	1320	44.19	6879	41.66	
Maternal depressive disorders before child’s death^c^					< 0.001
Yes	71	2.38	94	0.57	
No	2916	97.62	16,418	99.43	
Maternal chronic physical condition^d^					< 0.001
Yes	1060	35.49	1875	11.36	
No	1927	64.51	14,637	88.64	
Treatment seeking for depressive disorders after child’s death^e^					< 0.001
Yes	149	4.99	130	0.79	
No	2838	95.01	16,382	99.21	
**Paternal characteristics (*N*** **= 22,011)**					
Paternal age at child’s death					< 0.001
< 35 years	1338	31.42	6534	36.81	
≥ 35 years	2920	68.58	11,219	63.19	
Paternal depressive disorders before child’s death^c^					< 0.001
Yes	78	1.83	49	0.28	
No	4180	98.17	17,704	99.72	
Paternal chronic physical condition^d^					< 0.001
Yes	1650	38.75	2214	12.47	
No	2608	61.25	15,539	87.53	
Treatment seeking for depressive disorders after child’s death^e^					< 0.001
Yes	104	2.44	92	0.52	
No	4154	97.56	17,661	99.48	

^a^ Refers to insurance covered under the name of the child himself or herself or the name of a grandparent

^b^ In the year before the index child’s death

^c^ Depressive disorder (ICD-9-CM codes: 296.2X, 296.3X, 300.4X, and 311) is confirmed by receiving a diagnosis by a psychiatrist in (i) two or more outpatient services or (ii) one inpatient service within three years before the index child’s death

^d^ Chronic physical condition — including malignant neoplasms, chronic liver disease and cirrhosis, heart disease, cerebrovascular disease, diabetes, hypertensive disease, bronchitis, emphysema, asthma, and atherosclerosis — were obtained from the three-year reimbursement records prior to the index child’s death

^e^ Depressive disorder (ICD-9-CM codes: 296.2X, 296.3X, 300.4X, and 311) is confirmed by receiving a diagnosis by a psychiatrist in (i) two or more outpatient services or (ii) one inpatient service in the three years after the index child’s death

With stratification by parents’ gender, the estimated hazard of seeking MDD treatment after three years of a young child loss was depicted in Fig. 1. The hazards of MDD were generally greater in mothers, regardless of bereavement status. For the bereaved mothers, the hazard fell within the first four months (see the solid lines of the left panel) and reached the platform near the 14th month. In contrast, the hazard of seeking MDD treatment for the bereaved fathers abruptly fell in the first eight months after losing a young child, remaining moderately fluctuated till the end of 22nd month (see the solid lines of the right panel).

For the middle-aged mothers, univariate Cox regression analyses indicated that having lost a child to death (HR = 6.54), having the prior history of MDD (HR = 65.1), and having a CPC (HR = 8.13) were the three strongest predictors for increased hazard of visiting a doctor for MDD (see Table 2). In addition, having a child who received a diagnosis of a catastrophic disease (e.g., cancer) may increase maternal hazard of receiving the treatment for MDD by 172% (less than one year) and 386% (one year or more), and disadvantaged income may also slightly increase such risk (poverty: HR = 2.32). With statistical adjustment for child’s health and family characteristics, losing a child was found to increase the hazard of receiving the treatment of MDD by 506% (Model I: 95% CI = 4.47–8.21), and such risk was slightly attenuated while maternal sociodemographic and health characteristics were taken into account simultaneously (Model II: aHR = 4.71; 95% CI =3.35–6.64).

**Table 2:** Child loss in relation to subsequent treatment of major depressive disorders in mothers (*N* = 19,499)

			Crude HR	***P***-value	Model I^**a**^		Model II^**a**^	
Variables	n	%			aHR	95% CI	aHR	95% CI
**Children’s characteristics**								
Death								
Yes	2987	15.32	6.54	< 0.001	6.06^***^	4.47–8.21	4.71^***^	3.35–6.64
No	16,512	84.68	1.00		1.00		1.00	
Diagnosis of catastrophic illness								
One year or more	1198	6.14	4.86	< 0.001	1.32	0.93–1.87	1.17	0.80–1.73
Less than one year	576	2.95	2.72	< 0.001	0.77	0.44–1.34	0.58	0.30–1.10
No	17,725	90.90	1.00		1.00		1.00	
**Family characteristics**								
Income level^b^								
High	5708	29.27	1.00		1.00		1.00	
Medium	9605	49.26	1.43	0.025	1.21	0.88–1.67	1.40	0.98–2.00
Poverty	3686	18.90	2.32	< 0.001	1.30	0.91–1.88	1.20	0.80–1.81
Urbanization^b^								
Urban	5655	29.00	1.00		1.00		1.00	
Suburban	12,124	62.18	1.31	0.068	1.33	0.99–1.78	1.36	0.99–1.88
Rural	1444	7.41	1.62	0.042	1.50	0.94–2.41	1.61	0.94–2.75
Linkage with father in health insurance								
Yes	12,105	62.08	1.00		1.00		1.00	
No	7394	37.92	1.33	0.021	1.00	0.77–1.29	0.98	0.70–1.36
**Maternal characteristics**								
Age at child’s death								
< 35 years	11,300	57.95	1.00				1.00	
≥ 35 years	8199	42.05	1.35	0.018			1.26	0.94–1.69
Premium payer (mother)^c^								
Yes	12,386	63.52	1.00				1.00	
No	6753	34.63	1.14	0.293			1.34	0.97–1.86
Depression disorders^d^								
Yes	165	0.85	65.1	< 0.001			39.2^***^	26.6–57.8
No	19,334	99.15	1.00				1.00	
Chronic physical condition^e^								
Yes	2935	15.05	8.15	< 0.001			3.72^***^	2.80–4.94
No	16,564	84.95	1.00				1.00	

^a^ Model I adjusted for children’s listed characteristics, gender, and age; Model II adjusted for children’s and maternal characteristics; ^***^P < 0.001; ^**^P < 0.01; ^*^P < 0.05

^b^ Within the year before the index child’s death

^c^ “No” refers to health insurance covered by the father, children themselves, and grandparents

^d^ Depressive disorder (ICD-9-CM codes: 296.2X, 296.3X, 300.4X, and 311) is confirmed by receiving a diagnosis by a psychiatrist in (i) two or more outpatient services or (ii) one inpatient service within three years before the index child’s death

^e^ Chronic physical condition — including malignant neoplasms, chronic liver disease and cirrhosis, heart disease, cerebrovascular disease, diabetes, hypertensive disease, bronchitis, emphysema, asthma, and atherosclerosis — were obtained from the three-year reimbursement records prior to the index child’s death

The profile of significant predictors for receiving the treatment for MDD in fathers was similar with those in mothers, although variation exists in the magnitude (Table 3). The crude hazard ratio of MDD associated with losing a child was 4.71 in fathers, and the hazard ratio linked with prior history of MDD was as high as 117.3. For the middle-aged men, having a child with a catastrophic illness for one year or more and disadvantaged incomes may accordingly increase the hazard of MDD treatment seeking by 215 and 86% (*p* < 0.01). With all listed variables adjusted simultaneously, losing a young child to death was found to moderately increase fathers’ risk of MDD by 93% (Model II: 95% CI = 1.27–2.95).

**Table 3:** Child loss in relation to subsequent treatment of major depressive disorders in fathers (*N* = 22,011)

			Crude HR	***P***-value	Model I^**a**^		Model II^**a**^	
Variables	N	%			aHR	95% CI	aHR	95% CI
**Children’s characteristics**								
Death								
Yes	4258	19.34	4.71	< 0.001	4.47^***^	3.15–6.34	1.93^**^	1.27–2.95
No	17,753	80.66	1.00		1.00		1.00	
Diagnosis of catastrophic illness								
One year or more	731	3.32	3.15	< 0.001	1.21	0.68–2.14	1.12	0.57–2.22
Less than one year	1646	7.48	2.83	< 0.001	0.98	0.64–1.52	1.13	0.68–1.87
No	19,634	89.20	1.00		1.00		1.00	
**Family characteristics**								
Income level^b^								
High	6265	28.46	1.00		1.00		1.00	
Medium	10,637	48.33	1.22	0.302	1.21	0.83–1.76	1.23	0.81–1.86
Poverty	4597	20.89	1.86	0.003	1.42	0.94–2.15	1.52	0.95–2.43
Urbanization^b^								
Urban	5786	26.29	1.00		1.00		1.00	
Suburban	13,743	62.44	1.18	0.353	1.12	0.79–1.58	1.29	0.86–1.92
Rural	2112	9.60	1.02	0.939	0.97	0.55–1.72	1.09	0.58–2.05
Linkage with mother in health insurance								
Yes	12,105	55.00	1.00		1.00		1.00	
No	9906	45.00	1.51	0.005	1.01	0.75–1.38	0.92	0.62–1.36
**Paternal characteristics**								
Age at child’s death								
< 35 years	7872	35.76	1.00				1.00	
≥ 35 years	14,139	64.24	1.39	0.047			0.98	0.68–1.43
Premium payer (father)^c^								
Yes	15,515	70.49	1.00				1.00	
No	6129	27.85	0.90	0.524			0.69	0.44–1.07
Depressive disorders before child’s death^d^								
Yes	127	0.58	117.3	< 0.001			56.0^***^	34.5–90.8
No	21,884	99.42	1.00				1.00	
Chronic physical condition^e^								
Yes	3864	17.55	7.91	< 0.001			4.07^***^	2.90–5.73
No	18,147	82.45	1.00				1.00	

^a^ Model I adjusted for children’s listed characteristics, gender, and age; Model II adjusted for children’s and paternal characteristics; ^***^P < 0.001; ^**^P < 0.01; ^*^P < 0.05

^b^ Within the year before the index child’s death

^c^ “No” refers to health insurance covered by the mother, children themselves, and grandparents

^d^ Depressive disorder (ICD-9-CM codes: 296.2X, 296.3X, 300.4X, and 311) is confirmed by receiving a diagnosis by a psychiatrist in (i) two or more outpatient services or (ii) one inpatient service within three years before the index child’s death

^e^ Chronic physical condition — including malignant neoplasms, chronic liver disease and cirrhosis, heart disease, cerebrovascular disease, diabetes, hypertensive disease, bronchitis, emphysema, asthma, and atherosclerosis — were obtained from the three-year reimbursement records prior to the index child’s death

**Fig. 2:**
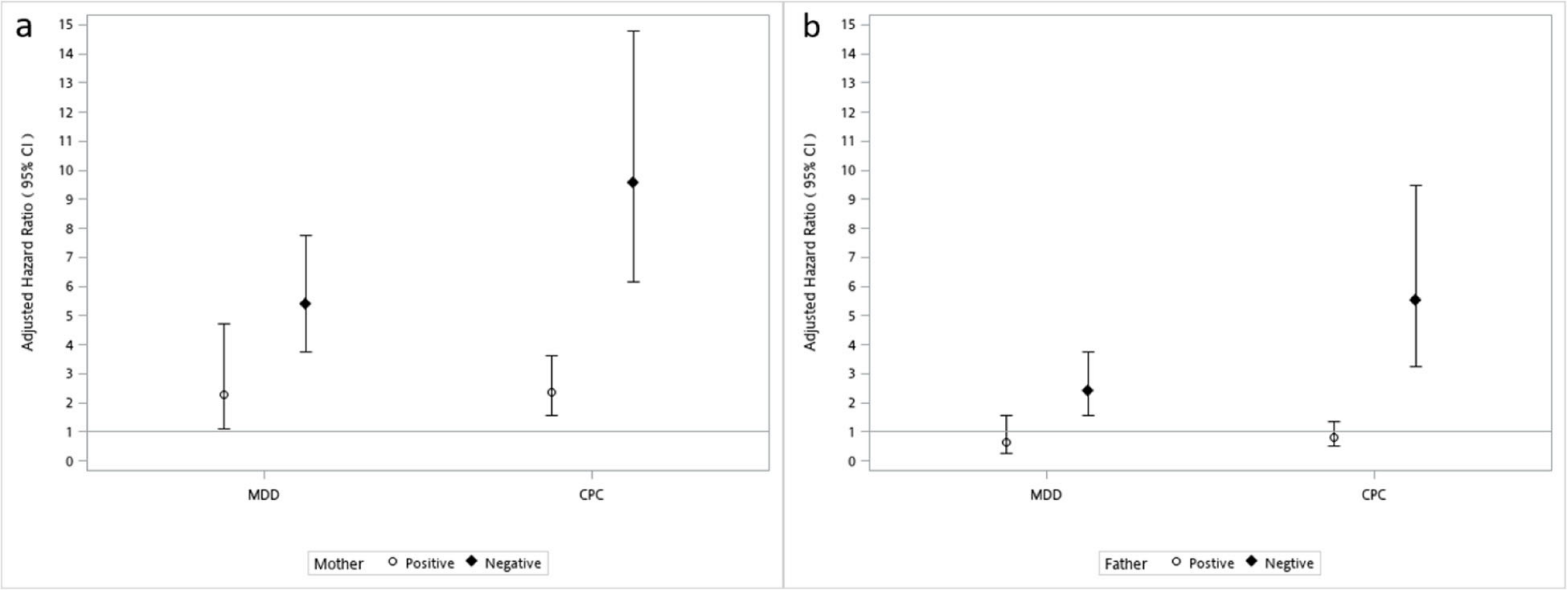
Adjusted hazard ratios of seeking treatment for major depressive disorders for the bereaved mothers and fathers, by prior history of major depressive disorders (MDD) and chronic physical condition (CPC). The interaction terms between losing a child and prior health condition were all statistically significant (p < 0.05) in both (**a**) mothers and (**b**) fathers

With a focus on risk variation in the subgroup defined by parents’ prior health condition, we found that the increased risk of MDD was especially prominent for those parents who received no medical treatment for chronic condition in the three years prior to the death of the index child (Fig. 2). A child’s death was associated with adjusted HR of 9.55 among those non-CPC affected mothers, whereas the estimate was only 2.38 for those who had at least one CPC. Indeed, for fathers, such increased hazard of bereavement effects was only significant for those who previously had no CPC (aHR = 5.54.06; 95% CI = 3.24–9.50) and had no MDD (aHR = 2.42; 95% CI = 1.55–3.78).

## Discussion

We utilized population-based data to identify the pairs of young children and their parents to investigate the extent to which losing a young child to death may affect parents’ subsequent bereavement effects, as indexed by treatment seeking for MDD, in the middle-aged population and explore how the risk operates differentially by prior health condition. Our analyses showed that the risk of MDD was especially prominent in the first year after the death of a child. The occurrence of MDD was elevated by 4.71-fold in the bereaved mothers, more than two times higher than 1.93-fold in fathers. Notably, the so-called “bereavement effects” were not homogenous across individuals: the significant increased risk of seeking MDD treatment was more salient in those who had no prior MDD or those who had no CPC (e.g., diabetes and hypertensive disease), especially in fathers.

On the basis of the ICD-9 CM codes, our findings indicated that parents’ increased risk of MDD subsequent to a child loss may manifest far beyond six months [7, 35–38]. This observation may partly resonate with conceptualization of pathological bereavement (i.e., persistent complex bereavement disorder and prolonged grief disorder) in DSM-V and ICD-11 [39, 40]. Disturbed/complicated grief is distinct from depression and post-traumatic stress disorder, although the rates of comorbidity between these conditions are high and the duration can be longer than 6 to 12 months after the episode of loss [41–44]. We also noted that the observed “bereavement effects” in the first year were slightly greater, yet less salient after that, than those reported previously in other Western societies (e.g., Canada). This discrepancy may be partly due to the variation in (i) cultural or societal differences in clinical manifestation of MDD, (ii) perceived need in treatments seeking, and (iii) the nature of outcome indicators (e.g., MDD vs. antidepressant; incident vs. prevalent cases) [45–47].

Our results corroborate previous findings that mothers may suffer more than fathers while facing the loss of a child, in terms of bereavement responses [15, 20, 22, 48]. The mechanisms underlying the observed gender differences in seeking treatment for MDD may be attributed to a mixed result of vulnerability, stress coping, help seeking, and role expectation in childcare [22, 49]. For example, the loss of a child may pose a threat to the identity of bereaved parents, which is especially true for women. Since the majority of primary caregivers for children are women in this study context, this adjustment process can be challenging for mothers and even more difficult for those unemployed ones with limited social support or a poor social network [50–52]. On the other hand, fathers often adopt emotional inhibition, detachment, or denial (i.e., ignoring their grief) as coping strategies, leading to unawareness of or even unresponsiveness to psychological distress. What makes the grief process complicated in the study context is that men may encounter more cultural or societal barrier in seeking medical help for mental health problems [45, 47]. In addition, given that the fathers may be the only breadwinner in the families of deceased children (see Table 1 for the relatively lower employment rate for mothers), the bereaved fathers likely accommodate the child loss with more work (or income) in order to regain the order of life, leaving no time for grief or selfcare [53–55].

Although the relationship between child loss and parental adverse health condition is fairly well established, the magnitude of bereavement effects is potentially complex because of parents’ attributes [17, 22, 54]. Our study illuminates that the parental bereavement effect may vary by prior health condition; specifically, the increased risk of treatment seeking for MDD was notably pronounced in those without prior history of MDD or CPC. Three probable explanations may account for this phenomenon. First, although child loss is a traumatic event, child-rearing, especially for the kids with catastrophic illness, is often a long and even financially and emotionally demanding process [56, 57]. Some parents may be aware of psychological distress and seek treatment long before the child’s death, as indicated by the work reporting that parental utilization of antidepressants or anxiolytics was gradually elevated in the three years before they lost a child to diseases [22]. This possibility is further reinforced by our analyses showing that prior prevalence of MDD treatment among bereaved parents was 4–6-times higher than their comparison peers. Second, for parents with physical or mental health problems (e.g., MDD), a visit to a doctor can offer a great opportunity to call for medical attention to somatic problems or stress-related symptoms (e.g., adjusting medication or therapy); thus, subsequent risk of MDD treatment associated with child loss may be alleviated. This also implicated the efficacy of timely medical intervention for parental bereavement before a child’s death. Third, the observation may reflect the delay in treatment seeking and the rebound after loss of their child. Preoccupation with care work (especially for the severely ill children) may face time or financial constraints, increasing the barriers to accessing appropriate healthcare [53].

Children’s health condition has long been recognized as a source of stressful life events substantially shaping child-rearing adults’ mental health, which is particularly true when children were affected by chronic or catastrophic illness, such as cancer [56–58]. However, in our analyses, diagnosis of catastrophic illness, a proxy for expectedness or disease chronicity, was not statistically associated with the hazard of MDD treatment seeking after we took into listed covariates into account. Even granting the existing confounding effects of family and parental characteristics, the observation can be the results of a wide heterogeneity in the subgroup of “not having catastrophic illness”, in terms of cause of death and the time from the index hospital visit to death. Future research linking other datasets (e.g., death certificates) can be of help in delineating how the nature of child’s disease or death may affect the process of parental grief and bereavement in our study population.

Some potential limitations of the present study should be acknowledged before further interpretation. First, the definition of exposure (i.e., child’s death) was primarily identified from health insurance records (e.g., insurance withdrawal), therefore restraining our ability to further characterize the heterogeneity of child’s death (e.g., cause of death) [22, 59]. In addition, we only used treatment seeking for MDD as a proxy for bereavement responses which may not be representative of a broad array of mental health sequelae (e.g., anxiety disorders, substance use disorder, and suicidal behaviors). It is possible that the observed association linking a child’s death and parental MDD treatment seeking may be partially attributed to other unadjusted variables which cannot be ascertained in the NHIRD, including family- (e.g., wealth), parents- (e.g., educational attainment), child- (e.g., low birthweight), and parent-child domains (e.g., quality of relationship). Another issue involves specification of parent-child pairs in relation to health insurance status. The insurance premium payer is usually determined by several factors (e.g., labor force participation and salary), some of which also have been found as correlates for bereavement [5]. The differential linkage rate in mother-child pairs (i.e., 51% in the deceased vs. 70% in the living, see Supplementary Fig. 1) may possibly result in the underestimated risk of MDD for the bereaved parents. Finally, the sole reliance on health insurance data can be problematic when only variables relevant to reimbursement can be gathered. Further exploration of variables at the child-, parents-, and household-levels was not feasible in this study; they should be evaluated in future research to better understand possible mechanisms.

Notwithstanding the limits just described, the present study has several strengths. To date, our study represents one of just a few population-based studies conducted in non-Western societies to investigate so-called parental bereavement effects in one society characterized by the very low (if not the lowest) birth rates. The large sample size and three-year follow-up period granted us the opportunity to delineate the effects of a child’s death on fathers and mothers with a rather thorough perspective in risk variation in individual health condition. Societies or cultures with similar parent-child bonding may benefit from this study in framing the concept of parental bereavement, which is particularly urgent in the context wherein mental health awareness is relatively limited and the need in mental healthcare is often overlooked.

## Conclusions

Our findings provide compelling evidence for an association between losing a young child to death and increased MDD in the subsequent three years. Timely intervention to reduce psychological distress in parents who have a child died depends on recognition of reaction and adaptation (e.g., withdrawal from social interaction) by pediatric services providers in inpatient and emergency department or home visits [5, 38, 57, 60]. For many parents, particularly those in disadvantaged economic or social network groups, the intervention may also involve financial aid and social support (e.g., patient groups). Effective strategies addressing enduring bereavement may require family-based, integrated physical and mental healthcare, and even extended counseling service long before the death of a child (e.g., pediatric hospice or intensive care unit). Additional studies of follow-up service delivery of intervention for those bereaved parents or caregivers are warranted, especially those who have received little medical attention in the process of caring for a seriously ill child or limited mental health literacy.

## Supplementary Information

**Additional file 1 Supplementary Fig. 1. Supplementary Fig. 2.** Log-negative-log of the survival function by losing a child (death): mothers (upper panel) and fathers (lower panel). **Supplementary Fig. 3.** Schoenfeld residuals by losing a child: mothers (upper panel) and fathers (lower panel). **Supplementary Fig. 4.** Smoothed hazard estimates in the mothers (upper panel) and fathers (lower panel) by losing a child, with stratification by prior history of major depressive disorder (MDD) and chronic physical condition (CPC).

## Data Availability

Data are available from the National Health Insurance Research Database (NHIRD) published by the Taiwan National Health Insurance (NHI) Administration. Due to legal restrictions imposed by the government of Taiwan concerning the “Personal Information Protection Act”, data cannot be made publicly available. Requests for data can be sent as a formal proposal to the NHIRD.

## Abbreviations

aHR: adjusted hazard ratio
CPC: chronic physical condition
HR: hazard ratio
ICD-9-CM: International Classification of Diseases, Ninth Revision, Clinical Modification
MDD: major depressive disorders
NHIP: National Health Insurance Plan
NHIRD: National Health Insurance Research Database
