# Supplementary material for: Gender differences in the risk of depressive disorders following the loss of a young child: a nationwide population-based longitudinal study
Source: BMC Psychiatry. 2021 Aug 20;21:414. doi: 10.1186/s12888-021-03421-w (PMC8377956; doi:10.1186/s12888-021-03421-w)

**Supplementary materials**

Data for the present study came from the 1998-2013 National Health Institute Research Data (NHIRD), derived from the National Health Insurance Plan (NHIP) in Taiwan. The datasets used for this proposed study contain registration files, including “registry for beneficiaries (ID),” “registry for contracted medical facilities (HOSB),” “registry for medical personnel (PER),” “registry for board-certified specialists (DOC),” “original claims data” (e.g., inpatient expenditures by admissions (DD), ambulatory care expenditures by visits (CD), “details of ambulatory care orders (OO),” “details of prescriptions dispensed at contracted pharmacies (GD),” “details for inpatient orders (DO),” and “details of drugs (DRUG),” among others. To take into account possible influences on the precision of estimates due to some rare variables of interest (e.g., child and adolescent mental disorders or drug events), instead of using the readily available datasets (e.g., the data composed of 5% random sampling of national health enrollees), special requests will be made to gain access to the entire NHIRD with additional “catastrophic/rare illness (HV)”. Patients with catastrophic illness certificates are exempt from copayments under the NHI program. Application for catastrophic illness certificates requires sufficient medical records and an independent peer review process. Therefore, the database for catastrophic illness (HV) is valid in high-ranking in our study.

We obtained the NHIRD from 2002 to 2010 with a specific focus on the age group between 0 to 12 years old. The death of a child was initially collected from HV with death date (3,612), DD with death or against-advise-discharge date (4,429), CD and ID with ICD-9 coding of 10 leading causes of death among decedents aged 0-11 years (1. Certain conditions originating in the perinatal period, 2. Congenital malformations, deformations, and chromosomal abnormalities, 3. Accidents and adverse effects, 4. Malignant neoplasms, 5. Disease of heart (except hypertensive diseases), 6. Pneumonia, 7. Sudden infant death syndrome, 8. Assault (homicide), 9. Septicemia, 10. Cerebrovascular diseases) in children plus withdraw at the same time (3,643), respectively. The child would be excluded if there was any claim data after withdrawing or mismatching between ID and HV/DD/CD (758). There were 10,926 deceased children and 5,809,757 alive children aged 0-12 y/o identified during 2002-2010. We further excluded the children under 1 y/o (because medical treatments for severe or congenital diseases were often recorded in the mother’s healthcare insurance account before the infants had their own individual identification numbers for reimbursement purpose) and those who could not be linked to their parents (8.77% in deceased group and 3.52% in alive group). Finally, the deceased children in our study were 5,881 and a 1:4 matched control (paired by children’s gender and age) were 23,524 alive children.

We have three different groups of parents (insurance covered by only father, only mother, or father and mother alternatively) collected by the study due to the nature of NHIRD for insurance claims during studied period. There were 4,258 fathers (68% only fathers) and 2,987 mothers (54% only mothers) identified in the bereaved cohort and 17,753 fathers (39.5% only fathers) and 16,512 mothers (35% only mothers) in the paired cohort. The children covered by father or mother was usually related to financial benefit, but not marital or inhabitant status of the family and it could be changed yearly.

**Supplementary Figure 1**

**Supplementary Figure 2**

Log-negative-log of the survival function by losing a child (death): mothers (upper panel) and fathers (lower panel)

Mothers


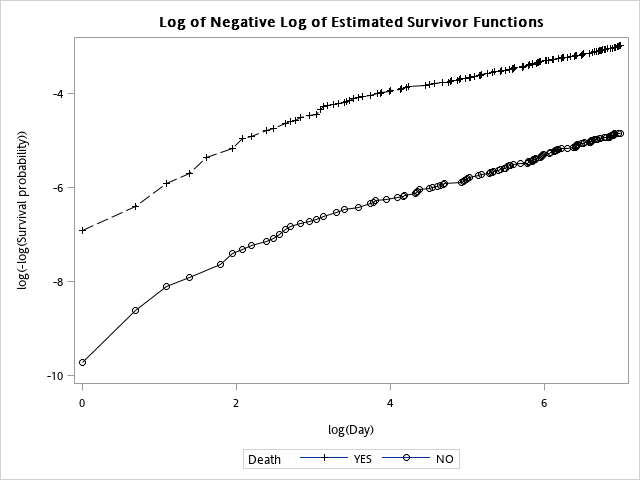


Fathers


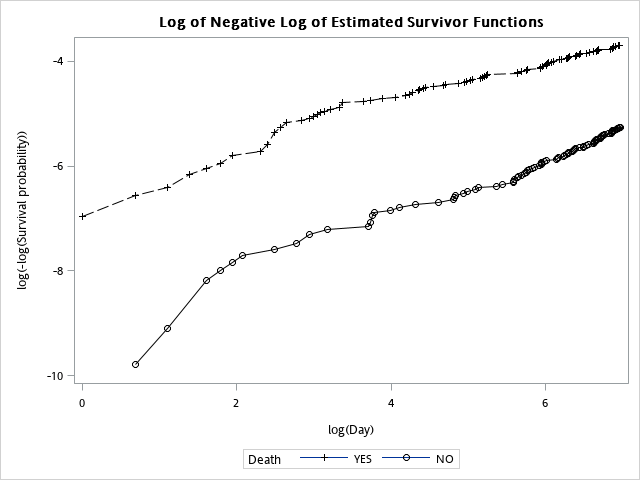


**Supplementary Figure 3**

Schoenfeld residuals by losing a child: mothers (upper panel) and fathers (lower panel)

Mothers


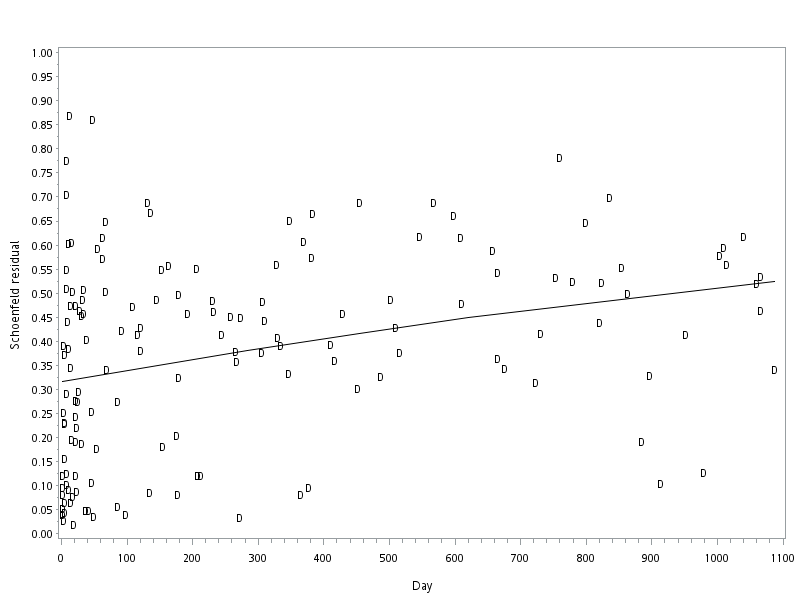


Fathers


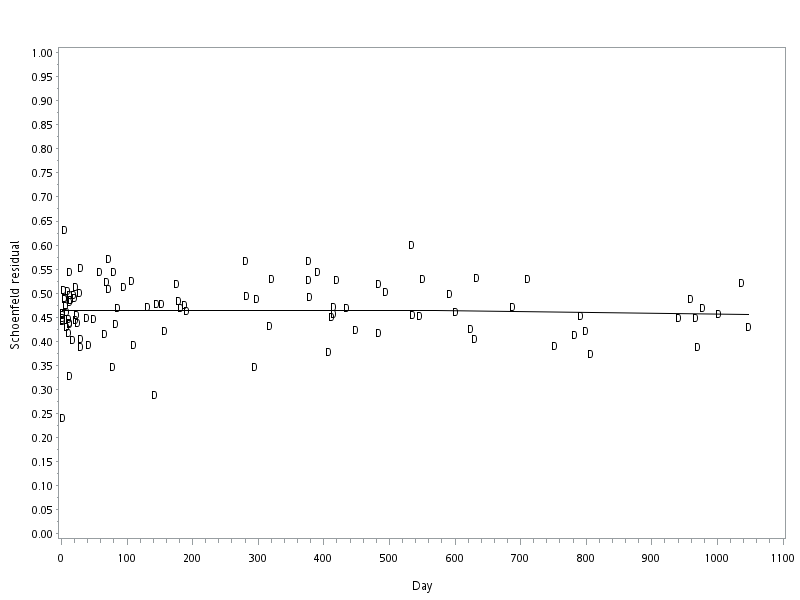


**Supplementary Figure 4**

Smoothed hazard estimates in the mothers (upper panel) and fathers (lower panel) by losing a child, with stratification by prior history of major depressive disorder (MDD) and chronic physical condition (CPC)

**Mothers**


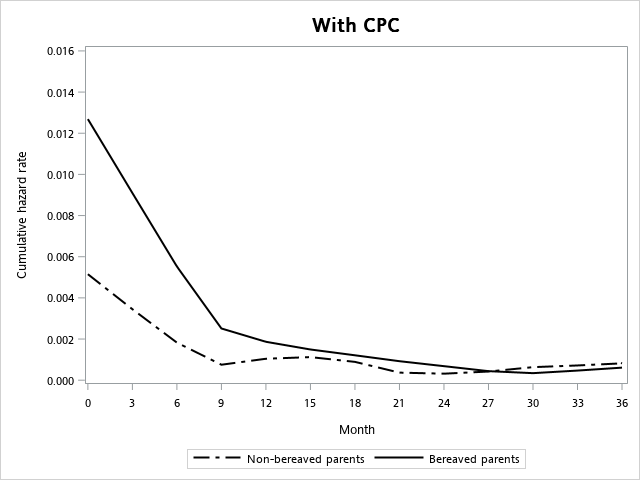

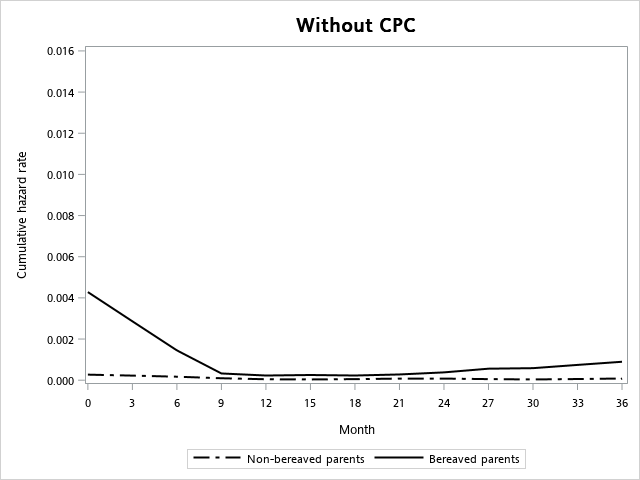

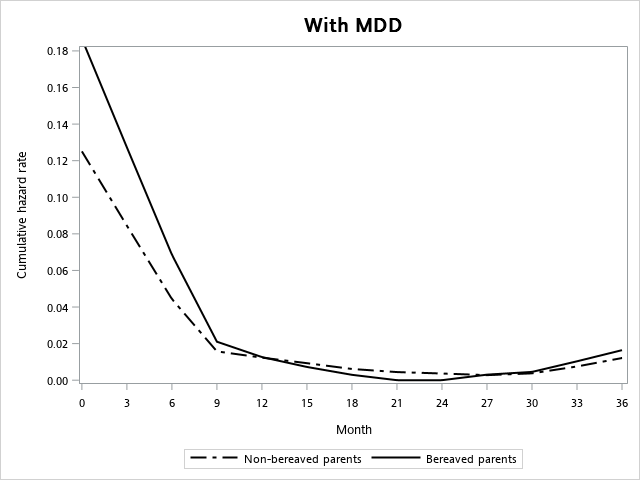

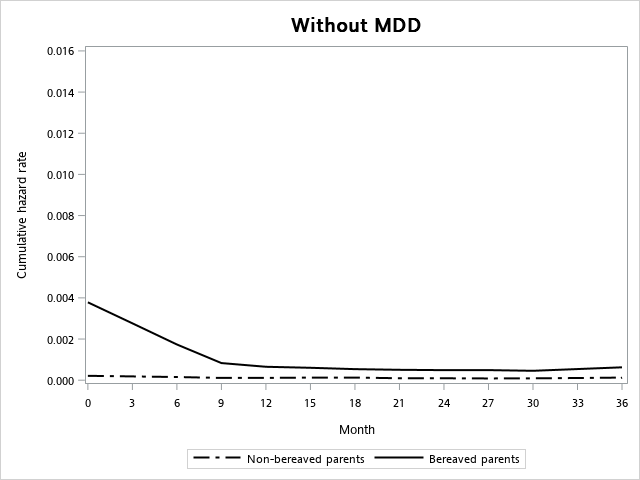


**Fathers**


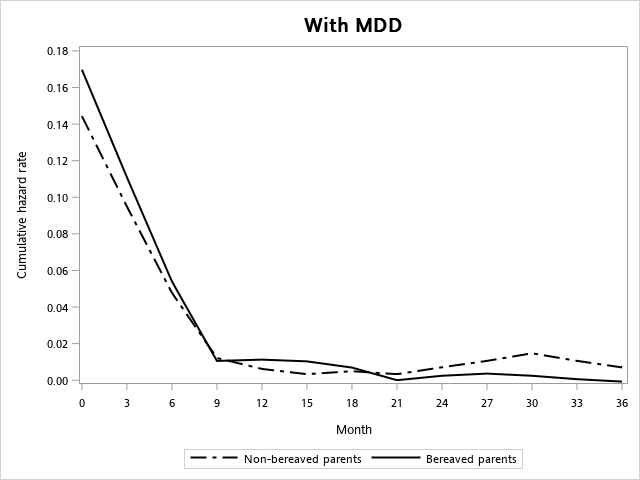

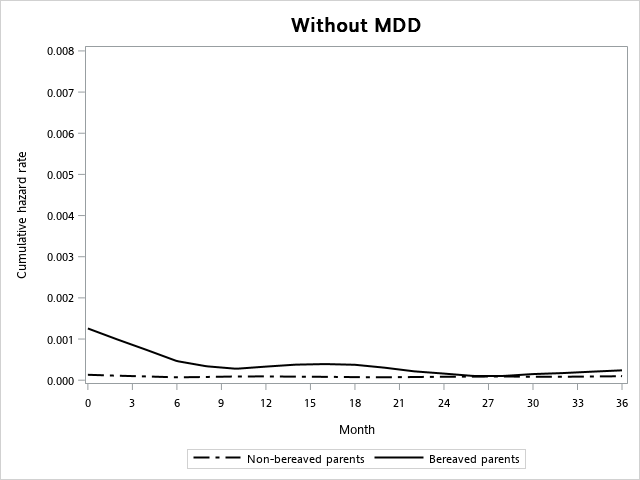

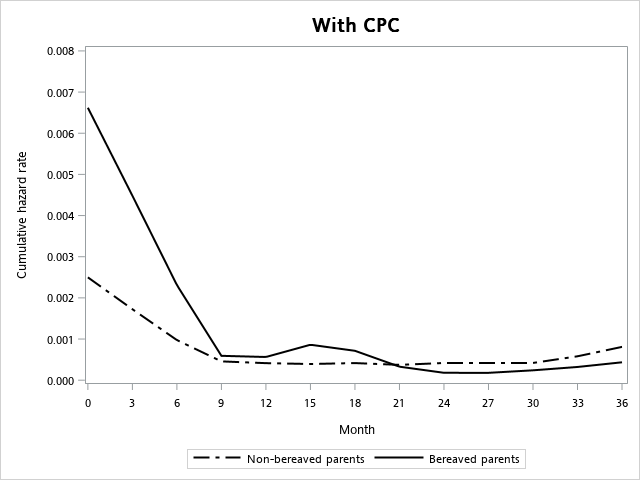

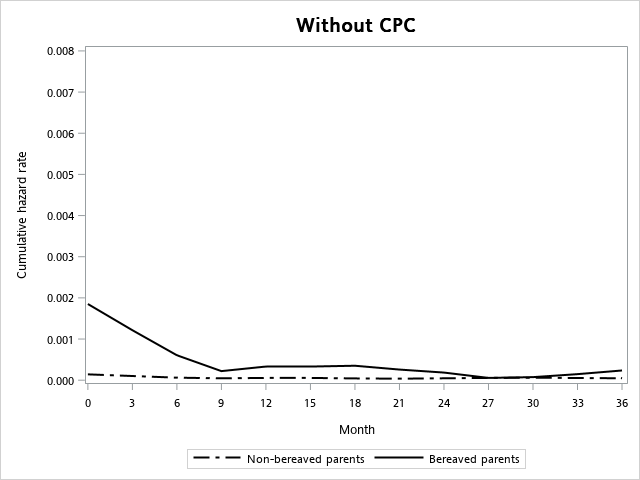

Supplement: Supplementary file 1 — Additional file 1 Supplementary Fig. 1. Supplementary Fig. 2. Log-negative-log of the survival function by losing a child (death): mothers (upper panel) and fathers (lower panel). Supplementary Fig. 3. Schoenfeld residuals by losing a child: mothers (upper panel) and fathers (lower panel). Supplementary Fig. 4. Smoothed hazard estimates in the mothers (upper panel) and fathers (lower panel) by losing a child, with stratification by prior history of major depressive disorder (MDD) and chronic physical condition (CPC). [file 12888_2021_3421_MOESM1_ESM.docx]
